# Supplementary material for: A Novel Method for Visualizing Melanosome and Melanin Distribution in Human Skin Tissues
Source: Int J Mol Sci. 2020 Nov 12;21(22):8514. doi: 10.3390/ijms21228514 (PMC7697890; doi:10.3390/ijms21228514)
Supplement: Supplementary file 1 [file ijms-21-08514-s001.pdf]

International Journal of
Molecular Sciences

Supplementary Materials

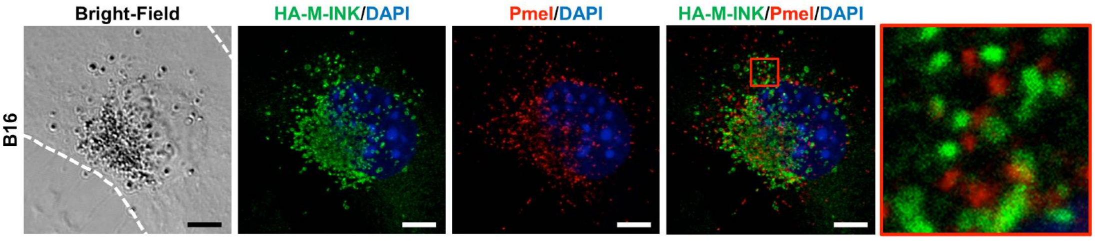

Figure S1. Immunostaining images of HA-M-INK and Pmel in B16 melanoma cells. Melanin-containing B16 melanoma cells were stained for HA-M-INK (green) and Pmel (red). DAPI was used as a nuclear counterstain (blue). The inset is a magnified view of the boxed area (red). The white dotted lines indicate the edge of the cell. Note that HA-M-INK-positive signals, representing black mature melanosomes, were poorly colocalized with Pmel-positive signals. Scale bars = 5 μm.
